# Does preoperative silodosin administration facilitate ureteral dilatation during flexible ureterorenoscopy? A randomized clinical trial

**DOI:** 10.1007/s11255-023-03824-6

**Published:** 2023-10-30

**Authors:** Tamer Diab, Waleed El-Shaer, Saad Ibrahim, Ehab El-Barky, Ahmed Abou Elezz

**Affiliations:** Urology Department, Faculty of Medicine, Benha University, Benha, Qalyubiyya Governorate Egypt

**Keywords:** Preoperative, Silodosin, Ureteral dilatation, Flexible ureterorenoscopy

## Abstract

**Purpose:**

To assess whether preoperative administration of silodosin can facilitate the placement of ureteral access sheath (UAS) prior to flexible ureteroscopy (F-URS) and reduce the occurrence of ureteric injury in challenging cases.

**Methods:**

This prospective randomized clinical trial was carried out on 147 patients diagnosed with upper ureteric stone or stone kidney, non-stented. The patients were randomly divided into two equal groups. Group A (silodosin group) included patients in whom F-URS was done with daily preoperative intake of 8 mg silodosin for 1 week and group B (placebo/control group) included patients in whom F-URS was done with daily preoperative intake of placebo tablets.

**Results:**

In group A, a total of 23 (33.3%) experienced ureteral wall injury following UAS insertion, while in group B, this occurred in 40 patients (59.7%). There was a statistically significant difference in the grade of ureteral wall injury between the two groups (*P* < 0.001). In the multiple regression analysis, age, operative time and silodosin were found to be significant risk factors for ureteral wall injury (*P* = 0.007, 0.041 and < 0.001, respectively).

**Conclusions:**

The administration of silodosin prior to retrograde intrarenal surgery (RIRS) effectively prevented significant ureteral wall damage and reduced initial postoperative discomfort.

## Introduction

Urolithiasis is a common urological disorder in the world, and it has a great effect on the global health system. The lifetime prevalence of urolithiasis is reported to be about 10%, and there is an estimated 50% recurrence of renal colic in 5 years after the first episode. It is a chronic disease with recurrent pain episodes, finally reaching the chronic kidney disease [1].

Since 1950s, technology for stone disease has been making us use new devices and change the management algorithm for every 10 years. The therapeutic goal is to achieve the highest possible stone-free rate (SFR) while minimizing invasiveness. Percutaneous nephrolithotomy and flexible ureterorenoscopy (f-URS) are the two most commonly used minimally invasive treatments for upper urinary tract stones [2].

The advancements in f-URS and accompanying equipment, such as the ureteral access sheath (UAS) and baskets, have greatly enhanced the benefits of f-URS procedures. These technological advancements have played a significant role in expanding the range of indications for f-URS procedures. In recent literature, it was reported that F-URS have 70–90% SFR with less complications and more tolerability [3].

However, application of f-URS is a complex multi-step act, and it may be difficult if any of consecutive actions fails during the operation. For instance, the ureteral orifice may not lead to the entrance and advancement of UAS; urethra, external urethral sphincter, prostate, and bladder neck may cause difficulty in UAS placement; and all these factors could complicate the f-URS procedure together [4].

The use of UAS insertion carries the risk of acute ureter damage and a higher likelihood of long-term ureter stricture. Although there is limited research on ureter injuries associated with UAS, one study reported an incidence rate of 46.5% for acute ureter wall damage. Furthermore, a significant proportion (up to 15%) of individuals with severe injuries showed involvement of the smooth muscle layer [5].

The lower portion of the ureter contains a significant number of α-adrenergic receptors (α-ARs), which play a role in the contraction of the ureteric smooth muscle when stimulated by phenylephrine. In certain instances, maneuvering the ureteroscope toward the stone becomes challenging or even impossible after passing through the ureteric orifice. In such cases, the insertion of a JJ stent is performed to dilate the ureter, and the ureterorenoscopy (URS) procedure is rescheduled for a later session [6].

Alpha-1 blockers, which are commonly used in the treatment of benign prostatic hyperplasia (BPH), have shown to be essential in medical expulsive therapy (MET), particularly for distal ureteral calculi. They help relax the muscles in the ureter, prevent spasms, and facilitate the dilation of the ureteral lumen [7].

The European Association of Urology recommends α-1-blokers for distal ureteral stones. Tamsulosin and silodosin are the main alpha1-blokers used for MET [8]. Silodosin selectively blocks α-1A 38 times higher than tamsulosin. In a study led by Gupta and co-worker, tamsulosin and silodosin caused 58% and 82% stone expulsion, respectively. Silodosin also had a shorter expulsion time than tamsulosin. Reversely thinking, the relaxation of ureter may facilitate an easy F-URS operation which can be achieved by using silodosin preoperatively [9].

We hypothesized that using silodosin preoperatively could ease the stages of the f-URS procedure via urethral, prostatic, bladder neck, and ureteral relaxation. We aimed to assess whether preoperative administration of silodosin can facilitate the placement of ureteral access sheath (UAS) prior to flexible ureteroscopy (F-URS) and reduce the occurrence of ureteric injury in challenging cases.

## Materials and methods

This prospective randomized parallel open label parallel clinical trial was carried out on 140 patients aged above 18 years old, both sex, and diagnosed with upper ureteric stone or stone kidney (with stone burden < 2 cm), non-stented and admitted to the Urology Department of Benha University Hospitals. The patients provided informed written consent before participating in the study. The research was conducted within the approved guidelines of the institutional ethical committee of Benha University Hospitals (Approval code: Ms 7–12-2022) during the period from March 2022 to March 2023. Additionally, the study was registered on clinicaltrials.gov (ID: NCT05798572).

The study excluded patients with the following conditions: refusal to participate; patients with acute or chronic renal insufficiency; pre-stented patients; those with uncorrected coagulopathy, previous open surgery (lumbar or ureteric), urinary tract infection, a history of alpha-blocker, PDF5 inhibitor and Ca-blocker medication (to avoid its effect on the degree of urethral dilatation), abnormal anatomies such as horseshoe kidney or duplicated ureter, suspected or known allergy to silodosin, and medication abuse. Also, for cases that exhibited failure application of UAS during the procedure and cases admitted immediately with severe pain, a double J stent was applied and then they were excluded from the research.

### Randomization

The patients were randomly allocated into two equal groups by a sealed opaque envelopes and a computer-generated sequence. Group A (silodosin group) included 73 patients for whom F-URS was done with daily preoperative intake of 8 mg silodosin for 1 week. Group B (Placebo/control group) included 74 patients for whom F-URS was done with daily preoperative intake of placebo tablets. The study was a double blinded, where the surgeon was blinded to whether the patient received silodosin or not and the statistician who arranged the data was also blinded to the procedures and the patients.

All patients were subjected to full history taking (age, sex, BMI) and physical examination was done on all the patients. Pre-interventional evaluation included general examination of the chest, heart and abdomen and vitals of the patient. Radiology investigation included pelvis abdominal ultrasonography, plain X-ray of the kidneys, ureters and bladder (KUB) and low-dose non-contrast computed tomography (NCCT). Laboratory investigation included urinalysis and culture and sensitivity if indicated. Routine preoperative laboratories included serum creatinine, serum urea, CBC, liver function tests and coagulation profile.

### Intervention evaluation

In group A (silodosin group), individuals received a daily dose of 8 mg of silodosin for 1 week prior to surgery. This dosage was determined based on the highest permitted dose for individuals with lower urinary tract symptoms. Patients who underwent the procedure while experiencing acute, unbearable pain despite medication were excluded from the study. Following admission and within 10 days of hospitalization, all patients received silodosin tablets for a week, underwent preoperative examination and surgery and were followed up. A prophylactic antibiotic was administered prior to the surgical procedure. The researcher assessed the adverse effects of silodosin and patient adherence to medication by counting the pills on the day of admission. In group B (Placebo/control group), patients underwent f-URS with daily preoperative intake of placebo tablets.

During the study period, a single expert surgeon (MSC), who had performed over 500 procedures for nephrolithiasis and conducted retrograde intrarenal surgery (RIRS) using an f-URS with a distal-end outer diameter of 8.5 Fr, performed the procedure on all patients.

### Technical procedures

All surgeries were performed with the patient under general anesthesia, in a lithotomy position. The surgeon routinely inserted a single safety guidewire and then positioned the UAS with the guidance of fluoroscopy. In all cases, a Navigator™ HD, with a length of 36 cm and a size of 12–14 Fr, was used to pass distal scopes in all the studied patients. The application was performed within normal movement (neither rapid nor slow), then it either passed smoothly, passed with resistance or did not pass (failed application). It was observed that in patients within the silodosin group, it passed smoothly, and few patients showed minimal resistance during passage and very few patients showed failure application of ureteral access sheath. In the control group, either passage was with resistance or failed application was observed. In case of failed application, a double J stent was applied to those patients and then they were excluded from the study. Following completion of the procedure, the UAS was removed while monitoring with ureteroscopy and for evaluation of the ureteral injuries. The entire surgical procedure was recorded using a video system, and any instances of ureteral wall injury were documented with serial-numbered pictures taken with a ureteroscope. The stones were treated by laser dusting. To minimize the effects of UAS (e.g., pain), all patients received a ureteral double J stent at the end of the operation. After UAS placement, operative notes including operative time, hospital stay and visual analog scale (VAS) measurements of pain severity (ranging from 0 for no pain to 10 for the worst possible pain) were recorded [10].

### Complication

After the completion of the surgery, another surgeon other than the operator performed a diagnostic ureteroscopy and visual record for evaluation and grading of the ureteral wall injury based on a previously established five-grade classification system. Grade 0 indicated no lesion or only mucosal petechiae, grade 1 represented mucosal erosion or a mucosal flap without smooth muscle injury, grade 2 involved both the mucosa and smooth muscle while sparing the adventitia, grade 3 denoted ureteral perforation encompassing the full thickness of the ureteral wall, including the adventitia, and grade 4 indicated complete ureteral avulsion. The procedure-related complications were assessed and categorized using the Clavien–Dindo classification system [11]. SFR was determined by Grade A (no fragments), Grade B (less than 2 mm) and Grade C (2–4 mm) [12]. The main focus of our study was to evaluate the severity of ureteral wall injury. Secondary outcomes included the occurrence of adverse events related to the medication, surgical complications, length of hospital stay and the rate of complete stone clearance. Additionally, all patients provided a subjective assessment of their postoperative pain using a visual analog scale (VAS) ranging from 0 (no pain) to 10 (severe pain), 3 h after the surgery. For patients with a VAS score of 5 or higher, active pain management was planned to use intravenous analgesics.

### Sample size

The sample size calculation was performed using G. power 3.1.9.2 (Universität Kiel, Germany). The sample size was calculated according to the grade of ureteral wall injury (our primary outcome) that was 9.3% in the silodosin group and 12 (27.3%) in the control group Based on 0.05 α error and 80% power of the study [13], the allocation ratio was 1:1. 13 cases were added to overcome dropout. Therefore, 147 patients were allocated.

### Statistical analysis

Statistical analysis was conducted using SPSS v27 (IBM^©^, Armonk, NY, USA). The normality of the data distribution was assessed using the Shapiro–Wilk test and histograms. Parametric data were reported as mean and standard deviation (SD) and analyzed using the unpaired Student’s *t* test. Using the Mann–Whitney test, nonparametric data were presented as median and interquartile range (IQR) and evaluated as median and IQR. When applicable, categorical variables were reported as frequency and percentage (percent) and evaluated using the Chi-square or Fisher's exact test. To investigate the link between the dependent and independent variables, multiple regression analysis was applied. A two-tailed *P* value < 0.05 was considered statistically significant.

## Results

**Fig. 1:**
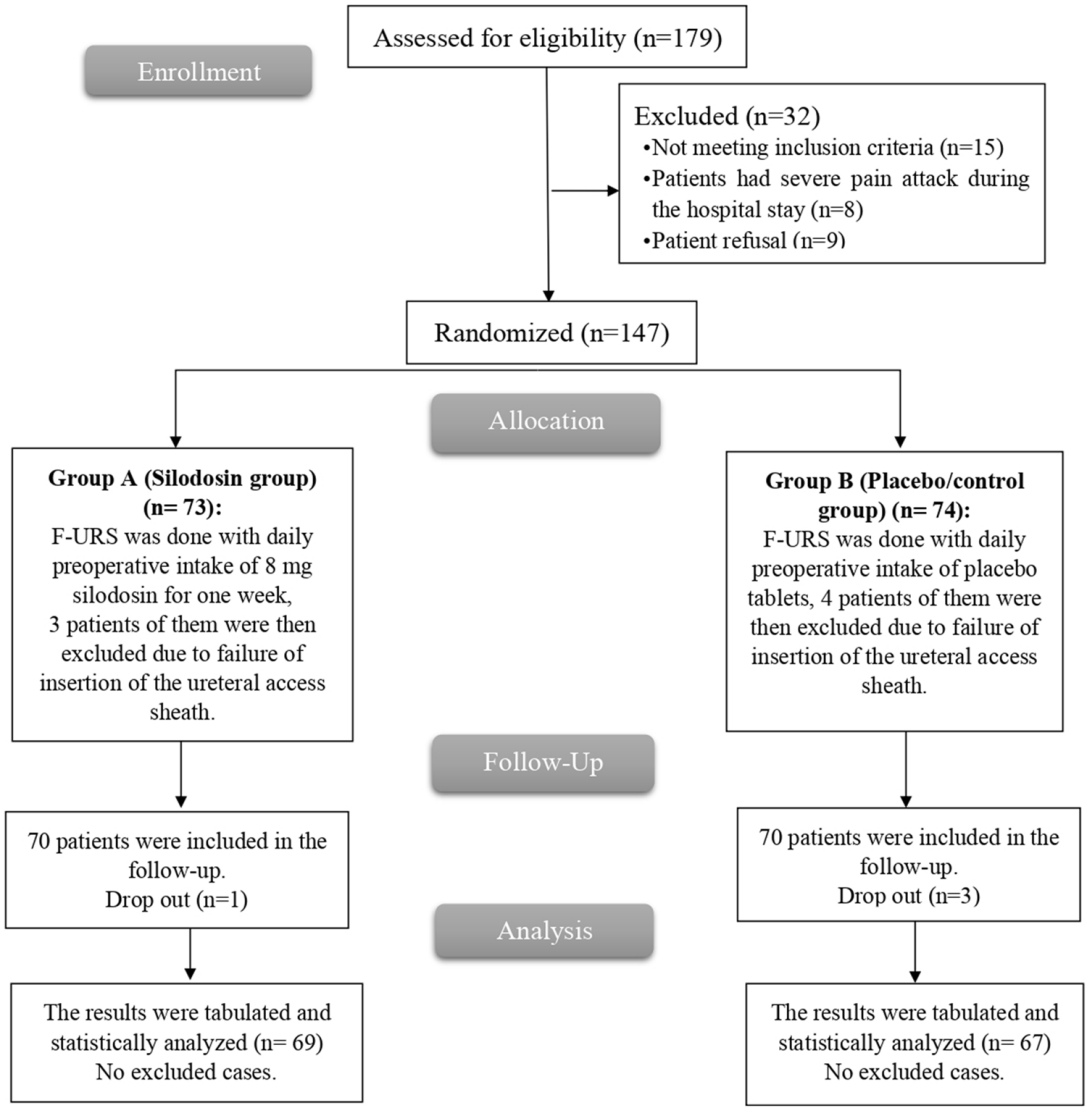
CONSORT flowchart of the enrolled patients

The study evaluated 179 patients for eligibility, with 15 patients failing to meet the criteria, 9 patients declining to participate and 8 patients had severe pain attacks during the hospital stay. The remaining 147 patients were divided into two groups of 73 patients in group A and 74 in group B, each through random allocation. Seven patients had difficulties during the ureter access sheath insertion, subjected to double J stenting and were also excluded. The remaining 140 patients were divided into two groups of 70 patients in group A and 70 in group B, each through random allocation. Four cases were lost during follow-up at 3 months (1 case in group A and 3 cases in group B). Then, 136 patients assigned to the groups were followed up and analyzed using statistical methods Fig. 1.

There were no significant differences observed between the two groups in terms of baseline characteristics such as age, sex, BMI and Charlson Comorbidity Index (Table 1).

**Table 1:** Baseline characteristics between the studied groups

	Group A (silodosin group) (*n* = 70)	Group B (control group) (*n* = 70)	*P* value
Age (years)	41.4 ± 14.26	42.4 ± 15.44	0.679
Sex			
Male	38 (54.29%)	41 (58.57%)	0.733
Female	32 (45.71%)	29 (41.43%)	
BMI (kg/m^2^)	26.9 ± 3.79	27.3 ± 3.97	0.602
Charlson Comorbidity Index			
0	32 (45.71%)	36 (51.43%)	0.640
1	20 (28.57%)	16 (22.86%)	
2	8 (11.43%)	11 (15.71%)	
> 3	10 (14.29%)	7 (10%)	

Data are presented as mean ± SD, median (IQR), or frequency (%)

*BMI* body mass index

There were no significant differences observed between the groups being studied in terms of stone characteristics (stone size and stone side) and stone Hounsfield unit (Table 2).

**Table 2:** Stone characters between the studied groups

	Group A (silodosin group) (*n* = 70)	Group B (control group) (*n* = 70)	*P* value
Stone size (mm)	12.5 ± 3.91	13 ± 3.71	0.479
Stone side			
Kidney stones	43 (61.43%)	39 (55.71%)	0.607
Upper ureteric stone	27 (38.57%)	31 (44.29%)	
Stone Hounsfield unit	1022 ± 259.6	1008.7 ± 266.4	0.765

Data are presented as mean ± SD or frequency (%)

*Statistically significant with *P* value < 0.05

Out of the patients in group A, 23 (33.3%) experienced ureteral wall injury following UAS insertion, while in group B, this occurred in 40 patients (59.7%). The presence of significant wall injury, classified as grade 2 or higher and involving an area above the smooth muscle layer, was detected in 6 patients (8.7%) in group A and in 26 patients (38.8%) in group B. There was a statistically significant difference in the grade of ureteral wall injury between the two groups (*P* < 0.001).

The VAS score was significantly lower in group A than in group B (3 vs. 5, *P* < 0.001). In terms of additional intravenous opioid analgesics administered to address intolerable pain, one patient in group A and four patients in group B required such treatment, but the difference between the groups was not statistically significant. The overall average operative time was significantly shorter in group A compared to group B (*P* = 0.040). There were no significant differences observed between the two groups in terms of hospital stay and stone-free rate.

In group A, 15 (21.7%) patients experienced grade I complications, while in group B, this occurred in 16 (23.9%) patients. Grade II complications were observed in 9 (13%) patients in group A and 16 (23.9%) patients in group B. Additionally, no patients in group A had grade IIIa complications, whereas 8 (11.9%) patients in group B experienced this type of complication. The occurrence of complications, as classified by the Clavien–Dindo system, showed a statistically significant difference between the two groups (*P* = 0.002). It is worth noting that in the non-silodosin group, patients exhibited clot retention that interfered with endoscopic evaluation, leading to a higher incidence of grade IIIa complications. Regarding the stone-free rate after the procedure, Class A was found in 23 (33.33%) patients in group A and 15 (22.39%)patients in group B, Class B was found in 46 (66.67%) patients in group A and 52 (77.61%) patients in group B and Class C not recorded in any patients in both groups (Table 3).

**Table 3:** Patients’ outcome between the studied groups

	Group A (silodosin group) (*n* = 69)	Group B (control group) (*n* = 67)	*P* value
Primary			
Grade of ureteral wall injury			
Low			< 0.001*
0	46 (66.7%)	27 (40.3%)	
1	17 (24.6%)	14 (20.9%)	
Significant			
2	4 (5.8%)	16 (23.9%)	
3	2 (2.9%)	10 (14.9%)	
Secondary			
VAS	3 (2–4)	5 (3–6)	< 0.001*
Analgesic requirement	1 (1.4%)	4 (6.0%)	0.205
Operative time (min)	64.4 ± 10.36	68.2 ± 11.6	0.040*
Hospital stay (day)	2 (2–3)	2 (2–4)	0.757
Complications (Clavien–Dindo)			
No	45 (65.2%)	27 (40.3%)	0.002*
G1	15 (21.7%)	16 (23.9%)	
GII	9 (13%)	16 (23.9%)	
GIII a	0 (0%)	8 (11.9%)	
Stone-free rate			
Class A	23 (33.33%)	15 (22.39%)	0.155
Class B	46 (66.67%)	52 (77.61%)	
Class C	0 (0%)	0 (0%)	

Data are presented as mean ± SD, median (IQR) or frequency (%)

*VAS* visual analog scale

*Statistically significant with *P* value < 0.05

In the multiple regression analysis, age, operative time and silodosin were found to be significant risk factors for ureteral wall injury (*P* = 0.007, 0.041 and < 0.001, respectively). However, no significant associations were observed between other stone-related factors and the occurrence of ureteral wall injury (Table 4).

**Table 4:** Multiple regression analysis of risk factors associated with ureteral access sheath-related significant ureteral wall injury

Independent variables	Coefficient	Std. error	*t*	*P*	*r*_partial_	*r*_semipartial_
Age (years)	0.011	0.004	2.730	0.007*	0.232	0.169
Age (≤ 55 years)	− 0.124	0.124	− 1.003	0.318	− 0.087	0.062
Sex	0.011	0.064	0.169	0.866	0.015	0.010
BMI (kg/m^2^)	− 0.014	0.011	− 1.308	0.193	− 0.113	0.081
Stone Hounsfield unit	− 0.000	0.0001	− 0.299	0.766	− 0.026	0.018
Stone size (mm)	− 0.003	0.010	− 0.321	0.749	− 0.028	0.020
Operative time (min)	0.006	0.003	2.064	0.041*	0.177	0.128
Silodosin	− 0.610	0.071	− 8.54	< 0.001*	− 0.598	0.528

*BMI* body mass index

^*^Statistically significant with *P* value < 0.05

## Discussion

Studies have provided evidence that alpha-blocker medications are safe and effective for both children and adults. Specifically, silodosin consistently demonstrates higher rates of stone expulsion, lower occurrence of adverse effects, reduced pain and decreased reliance on analgesics compared to calcium channel blockers, other adrenergic alpha-antagonists or placebo [14, 15]. Additionally, silodosin may have beneficial effects on stone-free rates and the duration of stone expulsion in both pediatric and adult populations [8].

Whenever possible, MET should be employed. However, it should be noted that spontaneous passage of the stone may not always be feasible, and surgical intervention may be required based on factors such as stone size, position and symptom progression. Specifically, stones larger than 5 mm or those located higher in the ureter are less likely to pass naturally. If the ureteroscope cannot pass through the ureter, there is a surgical risk, which may necessitate the placement of a stent and a subsequent operation once the ureter has adequately dilated [16].

In our study, we proposed the hypothesis that silodosin could potentially prevent significant ureteral wall injury associated with UAS insertion. To our knowledge, there is a lack of studies demonstrating the effectiveness of silodosin in reducing ureteral wall injury during UAS insertion.

According to the explanation, silodosin has the potential to be more effective than other alpha-blockers in dilating the ureter [17]. In the study conducted by Kim et al., it was found that premedication with silodosin resulted in reduced insertion force and decreased damage to the ureteral wall, even after 2–3 days of taking the medication. Moreover, since the therapy involves a single step, patients who received silodosin premedication experienced less pain associated with the stent and showed improved cost-effectiveness compared to those who underwent traditional stenting without the medication [13].

Within our study, we observed that out of the patients in group A, 23 (33.3%) experienced ureteral wall injury following UAS insertion, while in group B, this occurred in 40 patients (59.7%). The presence of significant wall injury, classified as grade 2 or higher and involving an area above the smooth muscle layer, was detected in 6 patients (8.7%) in group A and in 26 patients (38.8%) in group B. There was a statistically significant difference in the grade of ureteral wall injury between the two groups (*P* < 0.001).

Our findings revealed that silodosin reduced the intensity of postoperative pain in the early stages and also decreased the duration of surgery. We propose that by blocking α-adrenergic receptors, silodosin diminishes the strength of ureteral contractions and the frequency of ureteral peristalsis, resulting in a more relaxed and less active ureter. This relaxation allows for the utilization of a reinforced UAS, which, in turn, can generate significant shear stress, potentially leading to various levels of damage to the ureteral wall, ranging from mucosal erosion to ureteral avulsion [18].

Moreover, the interaction between the UAS and the mucosal layer triggers nociceptors, leading to rapid inflammatory responses and subsequent postoperative discomfort [19]. Therefore, silodosin has the potential to be more effective than other alpha-blockers in dilating the ureter. Experimental findings have shown that human ureters contain abundant messenger RNA for the a1D receptor, which includes all subtypes of a1A, a1B and a1D adrenoreceptors [20]. However, it is known that the a1A adrenoreceptor is primarily responsible for contraction of the human ureter [21]. According to reports, a1D receptors are mostly intracellular, whereas smooth muscle cell membranes carry a1A receptors [22].

Therefore, silodosin, a super selective a1A antagonist, may be significantly more effective in preventing ureter damage during UAS implantation [23]. The onset of action for silodosin seems to be faster compared to other alpha-blockers. In clinical practice, significant symptom relief was observed as early as the day following silodosin treatment, whereas tamsulosin took 4–7 days to alleviate symptoms. This suggests that silodosin may be beneficial in preventing ureteral wall injury, even with a short administration interval [24, 25].

This finding aligns with the results of Kim et al., who conducted a study involving 44 patients in the control group and 43 patients in the silodosin group. Their research demonstrated that prevention of significant postoperative ureteral injury that involved the smooth muscle layer was more successful with silodosin than in the control group (9.3 vs. 27.3%; *p* = 0.031). Additionally, patients who received silodosin prior to f-URS reported lower pain scores compared to those in the control group [13].

In a recent study conducted on an adult population, a higher concentration of alpha-adrenergic receptors in the distal ureter was utilized to facilitate the dilation of this segment of the ureter through the use of tamsulosin. The researchers postulated that administering -1A receptor agonists before surgery would decrease the failure rate of UAS passage during URS for stone removal. Patients who received tamsulosin treatment for at least 1 week before the procedure exhibited significantly higher success rates in the first attempt of UAS passage compared to those who did not receive any preoperative treatment (87% vs. 43%). This led to a reduction in the number of anesthesia exposures for patients and decreased operative time and costs for healthcare providers [26].

In patients with RIRS, ureteral stents are also linked to lower urinary tract symptoms, flank discomfort, postoperative sepsis and poor cost-effectiveness [27, 28]. Numerous studies have demonstrated that stent-related symptoms lead to complete or partial work disability in over 50% of patients and an 80% decrease in quality of life. Despite the advantages of allowing UAS placement, it is expected that only 8% of patients regularly make use of this option [29, 30].

In Kim et al.’s [13] study, it was found that premedication with silodosin was successful in facilitating UAS insertion by reducing the insertion force and preventing significant ureteral wall injury. This effect was observed even after 2–3 days of medication. Additionally, patients who received silodosin premedication reported lower stent-related symptoms and exhibited greater cost-effectiveness compared to patients who underwent other methods of ureteral access. These findings align with our own research. Köprü et al. [31] observed that preoperative use of silodosin facilitated only an insignificant positive effect on UAS placement failure and eased the F-URS procedure.

Our study had certain limitations, as it was conducted at a single center. Additionally, due to the subjective and multidimensional nature of the postoperative pain, the VAS score may not fully capture the complete pain experience. However, clinical decision making relied on the pain scale, which indicates the active efforts made by clinicians to manage and control pain.

Further randomized double-blind multicenter larger studies from other institutions for further confirmation of the efficacy of preoperative administration of silodosin therapy with long-term follow-up would be necessary soon.

## Conclusions

Administering silodosin medication prior to the RIRS procedure proved effective in preventing significant ureteral wall injury and reducing acute postoperative pain. Due to its rapid onset and high selectivity, using silodosin as a premedication may be a more favorable alternative to other alpha-blockers for ureteral access sheath placement.

## Data Availability

The data that support the results and conclusions of this study are available from the corresponding author upon reasonable request.
